# Associations between proton pump inhibitors and Alzheimer’s disease: a nested case–control study using a Korean nationwide health screening cohort

**DOI:** 10.1186/s13195-022-01032-5

**Published:** 2022-07-01

**Authors:** Hyo Geun Choi, Joo-Hee Kim, Ji Hee Kim, Eun Soo Kim, Ha Young Park, Kyueng-Whan Min, Mi Jung Kwon

**Affiliations:** 1 Department of Otorhinolaryngology-Head & Neck Surgery, Hallym University Sacred Heart Hospital, Hallym University College of Medicine, Anyang, 14068 South Korea; 2 Division of Pulmonary, Allergy, and, Critical Care Medicine, Department of Medicine, Hallym University Sacred Heart Hospital, Hallym University College of Medicine, 22, Gwanpyeong-ro 170 beon-gil, Dongan-gu, Anyang-si, Gyeonggi-do 14068 Republic of Korea; 3 Department of Neurosurgery, Hallym University Sacred Heart Hospital, Hallym University College of Medicine, 22, Gwanpyeong-ro 170 beon-gil, Dongan-gu, Anyang-si, Gyeonggi-do 14068 Republic of Korea; 4 Department of Radiology, Hallym University Sacred Heart Hospital, Hallym University College of Medicine, 22, Gwanpyeong-ro 170 beon-gil, Dongan-gu, Anyang-si, Gyeonggi-do 14068 Republic of Korea; 5 Department of Pathology, Busan Paik Hospital, Inje University College of Medicine, Busan, Republic of Korea; 6 Department of Pathology, Hanyang University Guri Hospital, Hanyang University College of Medicine, Guri, Republic of Korea; 7 Department of Pathology (Division of Neuropathology), Hallym University Sacred Heart Hospital, Hallym University College of Medicine, 22, Gwanpyeong-ro 170 beon-gil, Dongan-gu, Anyang-si, Gyeonggi-do 14068 Republic of Korea

**Keywords:** Alzheimer’s disease, Dementia, Nested case–control study, Proton pump inhibitors

## Abstract

**Background:**

Safety concerns against the use of proton pump inhibitors (PPIs) based on the risk of dementia, especially Alzheimer’s disease (AD), remain controversial. Here, we investigated the likelihood of AD depending on previous PPI exposure, use duration, and PPI generation.

**Methods:**

This nested case–control study comprised 17,225 AD patients who were 1:4 matched with 68,900 controls for age, sex, income, and region of residence from Korean National Health Insurance Service-Health Screening Cohort data between 2002 and 2015 using propensity-score matching method. Conditional and unconditional logistic regression analyses were used to evaluate the effects of previous PPI use on AD adjusting for multiple covariates.

**Results:**

Prior PPI use increased likelihood for AD in current and past PPI users (adjusted odds ratio 1.36 [95% confidence interval (CI) = 1.26–1.46] and 1.11 [95% CI = 1.04–1.18], respectively). Participants with either < 30 days, 30–90 days, or > 90 days of PPI prescription showed higher odds for AD (1.13 [95% CI = 1.07–1.19]; 1.18 [95% CI = 1.10–1.27]; 1.26 [95% CI = 1.16–1.36], respectively). Participants with either 1st-generation or 2nd-generation PPIs demonstrated higher incidences of AD in those with < 30 days (1.14 [95% CI = 1.07–1.22] and 1.13 [95% CI = 1.05–1.22], respectively), 30–90 days (1.19 [95% CI = 1.09–1.30] and 1.17 [95% CI = 1.05–1.29], respectively), or > 90 days (1.18 [95% CI = 1.07–1.30] and 1.27 [95% CI = 1.14–1.43], respectively) of prescription.

**Conclusions:**

Prior PPI use, regardless of current or past exposure, duration of use, or use of 1st- or 2nd-generation PPIs, may increase likelihood of AD, providing supportive evidence of previous pharmacoepidemiologic studies.

**Supplementary Information:**

The online version contains supplementary material available at 10.1186/s13195-022-01032-5.

## Introduction

Alzheimer’s disease (AD) is the most common form of dementia, accounting for 75% of cases and mainly affecting elderly individuals [1]. AD is characterized by mixed proteinopathy of abnormal deposits of β-amyloid and tau protein in the brain, leading to neurodegenerative damage [2, 3]. It is a significant physical, emotional, and financial burden on patients, their families, and society [4, 5]. The Korean Dementia Observatory 2020 report indicated a continuous and rapid increase of 30% in the last decade from 2010 to 2019 with aging society [1]. The number of patients affected is expected to rise to over 3 million (16%) by 2050 from 10% of those over 65 years old in Korea in 2019 [1]. Due to the major burden on public health and the lack of curative medication, studies evaluating the neurologic adverse effects of commonly used medications in the elderly population are of great public health importance for dementia prevention.

Proton pump inhibitors (PPIs) are the first choice drugs for treating acid-related diseases and are the most potent inhibitors of gastric acid secretion [6]. These agents suppress gastric acid secretion by irreversible inhibition of H + /K + ATPase on gastric parietal cells [7]. Newer PPIs, such as rabeprazole, ilaprazole, and astemizole, known as 2nd-generation PPIs, are more stable and potent at inhibiting acid secretion than first-generation PPIs (omeprazole, lansoprazole, and pantoprazole) [6]. With the increasing use of PPIs, safety issues regarding PPIs have been raised, and dementia events are one of the major concerns. The association between PPI and dementia is potentially mediated by a PPI-induced increase in the abnormal protein β-amyloid that contributes to the pathophysiology of AD [8, 9] or vitamin B_12_ deficiency caused by malabsorption induced by PPIs in animal and cell models [10].

Epidemiological research has demonstrated that PPI use could increase dementia events [11–14], raising concerns of the risk of dementia or cognitive impairment in elderly populations. However, there have been conflicting conclusions regarding the association between PPI use and dementia, specifically AD [15]. Some studies found no significant association between PPIs and dementia [16–19]. In other studies, the use of PPIs was beneficial in preventing dementia in the elderly [20, 21].

Since evaluating the risk of PPIs on dementia as a whole may mask their effect on AD [22], we designed this study specifically to focus on the impact of PPIs on likelihood for AD. Accordingly, using nationwide Korean National Health Insurance Service-Health Screening Cohort data (KNHIS-HSC), we sought to examine the association between the previous use of PPIs and the risk of dementia in patients with AD compared to a matched control group.

## Methods

### Study population and participant selection

The ethics committee in Hallym University (2019–10-023) approved this study and permitted the requirement for written informed consent to be waved. This current study used the Korean National Health Insurance Service-Health Screening Cohort (KNHIS-HSC) database that provides population-based data on a representative stratified random sample cohort of Korean population for research purposes. The KNHIS is a mandatory nationwide health insurance policy in Korea and has covered medical support to more than 98% of all Korean citizens from 1999. Medical data were available as part of the insurance claim and included diagnosis, comorbidities, medications, and date of visit. The data files and all individuals’ information obtained from the KNHIS-HSC database were de-identified by scrambling the identification codes and were entirely anonymous. The diagnostic codes used in the KNHIS-HSC database follow the International Classification of Diseases, 10th Revision, Clinical Modification (ICD-10-CM). A detailed description of the KNHIS-HSC data is described previously [23].

We conducted a retrospective cohort study based on nested case–control design to assess the effect of PPI on people’s risk of subsequently developing AD, with two cohort groups: a PPI user group and a comparison group. A total of 20,087 AD participants at baseline were initially recruited from 514,866 adults aged above 40 with 615,488,428 medical claim codes at a minimum of two clinic visits from 2002 to 2015. AD was identified using ICD-10 codes G30 (Alzheimer’s disease) or F00 (dementia in Alzheimer’s disease) and ≥ 2 times with treatment histories. Exclusion criteria from AD participants was under 60 years old (*n* = 682), diagnosed in 2002 (1-year wash-out period, *n* = 168), missing records of body mass index (BMI), fasting blood glucose, or total cholesterol (*n* = 19).

Control group initially enrolled participants who were not diagnosed with AD (G30 or F00) from 2002 to 2015 (*n* = 494,779), with a random order selection to avoid selection bias. The control participants were excluded if they had been ever assigned with AD (G30 or F00) once (*n* = 5404).

**Fig. 1:**
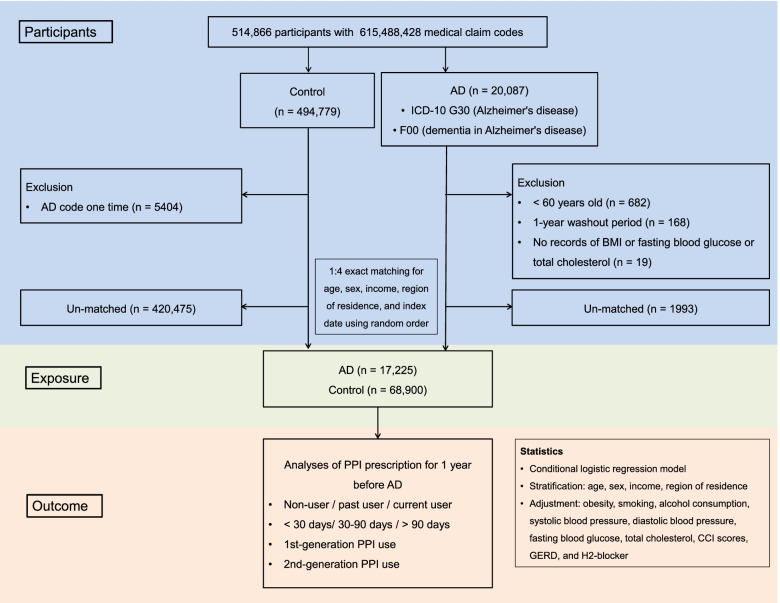
A schematic illustration of the participant selection process that was used in the present study. Of a total of 514,866 participants, 17,225 AD participants were matched with 68,900 control participants for age, sex, income, and region of residence

To optimize the balance of the AD and comparison groups’ baseline characteristics, a propensity score-matching was accessed on the basis of age, sex, income, and region of residence. The index date of each AD patient was determined as the day when the ICD-10 codes for AD (G30 or F00) were electronically assigned to patient in health insurance claims data sets. The index date of control participants followed along the index date of their matched AD participants. Through the matching steps, 1993 AD and 420,475 control participants were unmatched and excluded. Ultimately, 17,225 AD participants were matched with 68,900 control participants (Fig. 1). And then, we retrospectively reviewed the PPI prescription duration for 1 year before the diagnosis of AD in two cohort groups.

### Exposure (use of PPI)

KNHIS-HSC data offer information on prescription drugs including drug code, drug name, date of prescription, daily dose, and period. We gathered the prescription data of each participant for PPIs (lansoprazole, omeprazole, rabeprazole, pantoprazole, ilaprazole, esomeprazole, and dexlansoprazole). The days of PPI use were defined as the total prescription days during the 1 year before the index date [24]. The prescription of PPI was classified as nonuser, current PPI use (prescribed at least once within the previous 30 days), and past PPI use (prescribed at least once within the previous 31–365 days). The duration of total PPI use and the duration of PPI use depending on the generation were categorized as nonuser, < 30 days, 30 to 90 days, and > 90 days.

### Outcome (Alzheimer’s disease)

AD was defined based on ICD-10 code of G30 (Alzheimer’s disease) or F00 (dementia in Alzheimer’s disease). For the accuracy of diagnosis, we selected only the participants who had been treated ≥ 2 times [25, 26].

### Covariates

Ten age levels based on 5-year intervals and 5 income groups (class 1 [lowest income] to class 5 [highest income]) were categorized. The region of residence stratified as urban and rural areas, tobacco smoking, alcohol consumption, and obesity using BMI (kg/m^2^) were followed by previous study [27, 28]. The records of total cholesterol (mg/dL), diastolic blood pressure (DBP, mmHg), systolic blood pressure (SBP, mmHg), and fasting blood glucose (mg/dL) were measured. Charlson Comorbidity Index (CCI) score was used quantifying disease burden using major 17 comorbidities and the CCI scores calculated for these comorbidities were summed as the continuous variable (0 [no comorbidities] to 29 [multiple comorbidities]) [29]. Regarding PPIs, the number of patients diagnosed with gastroesophageal reflux disease (GERD) (ICD-10 code K21, treated ≥ 2 times and prescribed a PPI for ≥ 2 weeks) and the days of H2-blocker prescription were additionally assessed for the 1 year prior to the index date.

### Statistical analyses

Propensity score matching measured by logistic regression on the aforementioned baseline covariates was performed to minimize the difference between cohort groups using greedy option of nearest-neighbor matching algorithm [30]. Balance between groups was checked based on absolute standardized differences of covariates before and after matching. An absolute standardized difference of < 0.20 indicates good balance for a particular covariate [31]. Categorical data were summarized with numbers and percentages. Continuous data were depicted as mean and standard deviation. Characteristics between the AD and control groups were compared using the chi-square test for categorical variables and the independent *t*-test or one-way analysis of variance (ANOVA) for continuous variables.

To analyze the odds ratios (ORs) with 95% confidence intervals (CIs) for AD regarding PPI use, conditional logistic regression analysis was applied; crude (simple), model 1 (adjusted for SBP, DBP, fasting blood glucose, and total cholesterol), model 2 (adjusted for model 1 plus obesity, smoking, alcohol consumption, and CCI scores), and model 3 (adjusted for model 2 plus GERD and H2-blocker) were calculated. Subgroup analyses were performed by covariates. Two-tailed analyses were performed, and significance was defined as *P* values less than 0.05. SAS version 9.4 (SAS Institute Inc., Cary, NC, USA) was used for statistical analyses.

## Results

### Baseline characteristics of the study participants

This study comprised eligible 17,225 AD patients and 689,000 control participants. Because the AD and control groups were exactly matched, the demographic characteristics (age group, sex, economic level, and region of residence) were identical in both AD and control groups (standardized difference = 0). Except CCI values, other characteristics were similar between AD and control groups (standardized difference ≤ 0.2). The demographic and clinical characteristics are summarized in Table 1.

**Table 1:** General characteristics of participants

Characteristics		Total participants		
		AD	Control	Standardized difference
Total number (*n*, %)		17,225 (100.0)	689,000 (100.0)	
Age (years old) (*n*, %)				0.00
60–64		982 (5.7)	3928 (5.7)	
65–69		2268 (13.2)	9072 (13.2)	
70–74		4312 (25.0)	17,248 (25.0)	
75–79		5364 (31.1)	21,456 (31.1)	
80–84		3686 (21.4)	14,744 (21.4)	
85 +		613 (3.5)	2452 (3.5)	
Sex (*n*, %)				0.00
Male		6806 (39.5)	27,224 (39.5)	
Female		10,419 (60.5)	41,676 (60.5)	
Income (*n*, %)				0.00
1 (lowest)		3513 (20.4)	14,052 (20.4)	
2		1949 (11.3)	7796 (11.3)	
3		2320 (13.5)	9280 (13.5)	
4		3091 (17.9)	12,364 (17.9)	
5 (highest)		6352 (36.9)	25,408 (36.9)	
Region of residence (*n*, %)				0.00
Urban		6006 (34.9)	24,024 (34.9)	
Rural		11,219 (65.1)	44,876 (65.1)	
Obesity (*n*, %)^a^				0.10
Underweight		933 (5.4)	2975 (4.3)	
Normal		6921 (40.2)	25,135 (36.5)	
Overweight		4082 (23.7)	17,387 (25.2)	
Obese I		4795 (27.8)	21,168 (30.7)	
Obese II		494 (2.87)	2235 (3.24)	
Smoking status (*n*, %)				0.04
Nonsmoker		13,612 (79.0)	54,635 (79.3)	
Past smoker		1725 (10.0)	7452 (10.8)	
Current smoker		1888 (11.0)	6813 (9.8)	
Alcohol consumption (*n*, %)				0.08
< 1 time a week		13,381 (77.7)	51,270 (74.4)	
≥ 1 time a week		3844 (22.3)	17,630 (25.6)	
Systolic blood pressure (*n*, %)				0.03
< 120 mmHg		3780 (21.9)	14,442 (21.0)	
120–139 mmHg		8184 (47.5)	33,688 (48.9)	
≥ 140 mmHg		5261 (30.5)	20,770 (30.1)	
Diastolic blood pressure (*n*, %)				0.03
< 80 mmHg		7603 (44.1)	31,022 (45.0)	
80–89 mmHg		6177 (35.9)	24,947 (36.2)	
≥ 90 mmHg		3445 (20.0)	12,931 (18.8)	
Fasting blood glucose (*n*, %)				0.12
< 100 mg/dL		9292 (53.9)	39,603 (57.5)	
100–125 mg/dL		5328 (30.9)	21,545 (31.3)	
≥ 126 mg/dL		2605 (15.1)	7752 (11.3)	
Total cholesterol (*n*, %)				0.04
< 200 mg/dL		9345 (54.3)	38,088 (55.3)	
200–239 mg/dL		5315 (30.9)	21,564 (31.3)	
≥ 240 mg/dL		2565 (14.9)	9248 (13.4)	
CCI score (*n*, %)				0.4
0		6254 (36.3)	38,304 (55.6)	
1		4099 (23.8)	13,152 (19.1)	
≥ 2		6872 (39.9)	17,444 (25.3)	
Gastroesophageal reflux disease (*n*, %)				0.02
Yes		3267 (19.0)	12,542 (18.2)	
No		13,958 (81.0)	56,358 (81.8)	
The days of H2-blocker use^b^ (mean, SD)		62.25 (98.16)	43.79 (81.92)	0.20
Exposure to PPI (*n*, %)				0.10
Current		1264 (7.34)	3588 (5.21)	
Past		1652 (9.59)	5873 (8.52)	
Duration of PPI use (*n*, %)				0.10
< 30 days		1867 (10.8)	6663 (9.7)	
30–90 days		1162 (6.7)	3885 (5.4)	
> 90 days		1116 (6.5)	3437 (5.0)	
Duration of PPI use (1st-generation) (*n*, %)				0.09
< 30 days		1325 (7.7)	4406 (6.4)	
30–90 days		772 (4.5)	2434 (3.5)	
> 90 days		591 (3.4)	1848 (2.7)	
Duration of PPI use (2nd-generation) (*n*, %)				0.06
< 30 days		972 (5.6)	3479 (5.1)	
30–90 days		530 (3.1)	1795 (2.6)	
> 90 days		468 (2.7)	1412 (2.1)	

*Abbreviations*: *AD* Alzheimer’s disease, *CCI* Charlson comorbidity index, *PPI* proton pump inhibitor, *SD* standard deviation

^a^Obesity (BMI, body mass index, kg/m.^2^) was categorized as < 18.5 (underweight), ≥ 18.5 to < 23 (normal), ≥ 23 to < 25 (overweight), ≥ 25 to < 30 (obese I), and ≥ 30 (obese II)

^b^The H2-blocker use was included in the analyses because PPI users may take with or without concomitant use of H2-blockers

### Associations between previous PPI use and AD

**Fig. 2:**
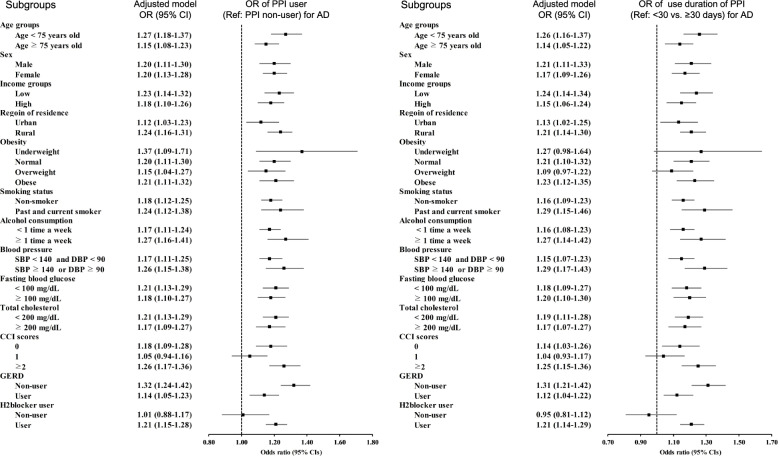
The odds ratios (95% confidence intervals) of previous proton pump inhibitor (PPI) exposure (*left panel*) and use duration of PPI (*right panel*) for AD based on subgroup analyses

We first examined the relationship between prior exposure to PPIs and AD compared to the control group (Table 2). The adjusted ORs were significant in both current and past PPI users before AD diagnosis ([1.36; 95% CI = 1.26–1.46; *P* < 0.001] and [1.11; 95% CI = 1.04–1.18; *P* < 0.001], respectively). PPI use remained associated with a higher possibility of AD in a subsequent subgroup analysis (*left panel* in Fig. 2 and Additional file 1: Supplementary 1).

**Table 2:** Crude and adjusted odd ratios of proton pump inhibitor (ref: non-user) for AD

Characteristics		*N* of AD	*N* of control	Odd ratios for AD (95% confidence interval)							
		exposure/total (%)	exposure/total (%)	Crude^†^	*P*-value	Model 1^†‡^	*P*-value	Model 2^†§^	*P*-value	Model 3^†§ ⁋^	*P*-value
Exposure to PPI											
Current		1264/17,225 (7.3%)	3588/68,900 (5.2%)	1.46 (1.37–1.57)	< 0.001*	1.46 (1.37–1.56)	< 0.001*	1.39 (1.30–1.49)	< 0.001*	1.36 (1.26–1.46)	< 0.001*
Past		1652/17,225 (9.6%)	5873/68,900 (8.5%)	1.17 (1.10–1.24)	< 0.001*	1.18 (1.11–1.25)	< 0.001*	1.14 (1.08–1.21)	< 0.001*	1.11 (1.04–1.18)	0.001*
Duration of PPI use											
< 30 days		1867/17,225 (10.8%)	6663/68,900 (9.7%)	1.18 (1.11–1.24)	< 0.001*	1.18 (1.12–1.25)	< 0.001*	1.17 (1.11–1.24)	< 0.001*	1.13 (1.07–1.19)	< 0.001*
30–90 days		1162/17,225 (6.7%)	3885/68,900 (5.6%)	1.26 (1.17–1.35)	< 0.001*	1.26 (1.18–1.35)	< 0.001*	1.23 (1.15–1.32)	< 0.001*	1.18 (1.10–1.27)	< 0.001*
> 90 days		1116/17,225 (6.5%)	3437/68,900 (5.0%)	1.36 (1.27–1.46)	< 0.001*	1.37 (1.27–1.47)	< 0.001*	1.28 (1.20–1.38)	< 0.001*	1.26 (1.16–1.36)	< 0.001*
Duration of PPI use (1st-generation)											
< 30 days		1325/17,225 (7.7%)	4406/68,900 (6.4%)	1.25 (1.17–1.33)	< 0.001*	1.25 (1.17–1.33)	< 0.001*	1.20 (1.12–1.28)	< 0.001*	1.14 (1.07–1.22)	< 0.001*
30–90 days		772/17,225 (4.5%)	2434/68,900 (3.5%)	1.31 (1.21–1.43)	< 0.001*	1.32 (1.22–1.44)	< 0.001*	1.26 (1.15–1.37)	< 0.001*	1.19 (1.09–1.30)	< 0.001*
> 90 days		591/17,225 (3.4%)	1848/68,900 (2.7%)	1.33 (1.21–1.46)	< 0.001*	1.33 (1.21–1.46)	< 0.001*	1.22 (1.11–1.34)	< 0.001*	1.18 (1.07–1.30)	0.001*
Duration of PPI use (2nd-generation)											
< 30 days		972/17,225 (5.6%)	3479/68,900 (5.1%)	1.14 (1.06–1.23)	< 0.001*	1.15 (1.06–1.23)	< 0.001*	1.18 (1.09–1.27)	< 0.001*	1.13 (1.05–1.22)	0.001*
30–90 days		530/17,225 (3.1%)	1795/68,900 (2.6%)	1.20 (1.09–1.33)	< 0.001*	1.21 (1.10–1.34)	< 0.001*	1.22 (1.11–1.35)	< 0.001*	1.17 (1.05–1.29)	0.003*
> 90 days		468/17,225 (2.7%)	1412/68,900 (2.1%)	1.35 (1.22–1.50)	< 0.001*	1.35 (1.22–1.50)	< 0.001*	1.31 (1.18–1.46)	< 0.001*	1.27 (1.14–1.43)	< 0.001*

*Abbreviations*: *AD* Alzheimer’s disease, *PPI* proton pump inhibitor

^*^Conditional logistic regression analysis, Significance at *P* < 0.05

^†^Stratified model for age, sex, income, and region of residence

^‡^Model 1 was adjusted for systolic blood pressure, diastolic blood pressure, fasting blood glucose, and total cholesterol

^§^Model 2 was adjusted for model 1 plus obesity, smoking, alcohol consumption, and Charlson Comorbidity Index (CCI) scores

^⁋^Model 3 was adjusted for model 2 plus gastroesophageal reflux disease (GERD) and H2-blocker

The H2-blocker use was included in the analyses because PPI users may take with or without concomitant use of H2-blockers

### Associations between duration of PPI use and AD

The ORs for AD were significantly increased regardless of the total prescription days of PPI in all 3 models (*P* < 0.001 for all; Table 2). Participants with either < 30 days, 30–90 days, or > 90 days of PPI prescription demonstrated higher odds for AD than those in the control group (1.13 [95% CI = 1.07–1.19, *P* < 0.001]; 1.18 [95% CI = 1.10–1.27, *P* < 0.001]; 1.26 [95% CI = 1.16–1.36, *P* < 0.001], respectively). We also observed an increased association between the cumulative duration of exposure to PPIs and developing AD. In most subgroup analyses, PPI users exposed for ≥ 30 days were associated with higher odds of AD than users exposed to < 30 days of use (*right panel* in Fig. 2 and Additional file 1: Supplementary 2).

### Associations between PPI generation and AD

**Fig. 3:**
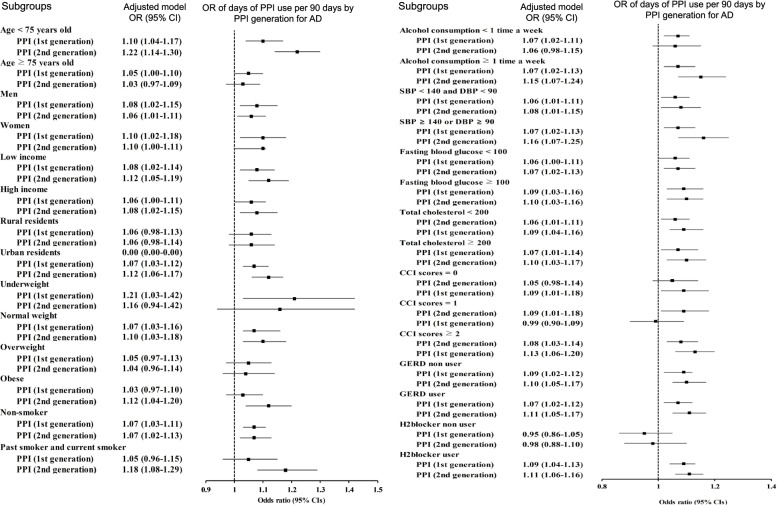
The odds ratios (95% confidence intervals) of PPI generations for AD based on subgroup analyses

We further examined the associations with the development of AD based on PPI generation, which was classified into 1st-generation and 2nd-generation PPIs. Both 1st-generation and 2nd-generation PPIs were associated with increased odds for AD regardless of the duration days in all 3 models (*P* < 0.005 for all; Table 2). Participants with either the 1st-generation or 2nd-generation PPIs demonstrated higher odds for AD in those with durations < 30 days (1.14 [95% CI = 1.07–1.22, *P* < 0.001] and 1.13 [95% CI = 1.05–1.22, *P* = 0.001], respectively), 30–90 days (1.19 [95% CI = 1.09–1.30, *P* < 0.001] and 1.17 [95% CI = 1.05–1.29, *P* = 0.003], respectively), or > 90 days (1.18 [95% CI = 1.07–1.30, *P* = 0.001] and 1.27 [95% CI = 1.14–1.43, *P* < 0.001], respectively). Subgroup analyses, which were conducted based on the use of 1st-generation or 2nd-generation PPIs, supported the observed effect of PPIs on AD (Fig. 3 and Additional file 1: Supplementary 3).

## Discussion

This nationwide nested case–control study indicated prior PPI exposure, regardless of current or past use, use duration, or use of 1st- or 2nd-generation PPIs, may increase the likelihood of AD in the > 60-year-old Korean population compared to the matched control groups. This negative impact of PPI use resulting in an increased odds for AD was maintained regardless of age, sex, income, region of residence, smoking, alcohol consumption, blood pressure, fasting blood glucose, total cholesterol, and GERD status. Our results highlight reminding the cautious and strict application of PPI medication following treatment guideline in order to prevent potential adverse effect of one of the most commonly used medication worldwide.

Despite an increasing number of studies demonstrating the effect of PPI use on risk of dementia, large-scale nationwide studies are limited to AD patients with a history of previous PPI use, and the effects of PPI use on AD are often conflicting. Our findings are in line with results from a prospective multicenter cohort study (AgeCoDe) based on a large population conducted in Germany [11]. Among 3327 persons aged ≥ 75 years, Haenisch et al. [11] showed that patients receiving PPI medication had a significantly increased risk of AD (hazard ratio 1.44; 95% CI = 1.01–2.06) and any dementia (1.38; 95% CI = 1.04–1.83) compared with nonusers. A recent community-based study in Spain reported an increased risk of AD (OR 1.47; 95% CI = 1.18–1.83) and non-AD dementias (1.38; 95% CI = 1.12–1.70) in users of two types of PPIs compared with those who used only one type of PPI [22], although the authors did not find a higher incidence of AD among the entire sample of PPI users. On the other hand, a prospective population-based study did not find any relations between exposure or duration of PPI use and increased risk of possible or probable AD [16]. However, this study was conducted with volunteers who had at least one follow-up visit for 10 years. Those participants included in the study were likely to care more about their health care and might take more medications than nonparticipants; hence, the generalizability seems limited. A recent study using Taiwan’s health insurance database reported no association between PPI use and AD in older adults [17]. The number of patients with AD (*n* = 428) and controls (*n* = 1712) were much smaller than those in the present study (17,225 people with AD and 689,000 controls). To avoid selection bias and heterogeneity of the study, we used a methodologically preferable study design using nationwide population-based controls and comprehensively considered the possible confounders. The majority of previous studies that concluded no association of PPI use with dementia did not consider the dementia type in their analysis [18, 32–34]. In our study comprising a nationwide cohort, we were able to reproduce the finding of the potential link between previous PPI use and AD.

Possible explanations of the involvement of PPIs in AD may find clues in experimental studies [8, 35]. Acidification of lysosomes determines the ability of microglia to degrade β-amyloid [8]. PPIs penetrate the blood–brain barrier in animals and can inhibit vacuolar-type H^+^–adenosine triphosphatase proton pumps of lysosomes [8, 9, 36], which suppresses the acidification of lysosomes [35]. As a result, PPIs may contribute to the inhibition of acidification, reduced β-amyloid degradation, and enhanced β-amyloid deposition. Of note, PPIs had a greater effect on AD risk in patients using concurrent H2-blockers in the present study, lending support to the theory that blocking acidification may be driving β-amyloid deposits in the brain. PPIs are consumed for long periods in conditions such as GERD, with the resultant exposure of the human brain to a substantial amount of PPIs [37]. Chronic consumption of PPIs may thus be a risk factor for AD [37]. Interestingly, short-term lansoprazole treatment in wild-type and AD transgenic mice dramatically increased β-amyloid levels in a dose-dependent manner [9]. Microglia treated with an ammonia pulse wash for 72 h were able to degrade a significant amount of β-amyloid in a single day [8], indicating that artificial lysosomal acidification is capable of affecting the amount of β-amyloid in the acute phase. In addition, PPIs have high binding and selective affinity for misfolded tau protein [38], indicating that PPIs may have a potential effect in the formation of neurofibrillary tangles of aggregated tau protein in addition to β-amyloid as the pathologic hallmarks of AD [2, 3]. In vitro and in vivo studies have shown that the sulfoxide scaffold found in PPIs has inherent affinity to neurofibrillary tangles in AD and related disorders (e.g., dementia with Lewy bodies and frontotemporal degeneration syndrome) [39]. Furthermore, recent experimental studies have demonstrated that PPI are potent and selective inhibitors of the acetylcholine-biosynthesizing enzyme, choline acetyltransferase, of which cholinergic dysfunction may cause major dementia disorders in the central nervous system [40] as well as infertility in the spermatic cholinergic system [41]. Nonetheless, clinical trials have failed to show similar adverse effects in dementia [33]. In a placebo-controlled randomized clinical trial (ClinicalTrials.gov number: NCT01776424), the PPI intake group did not show a significant difference in cognitive function when used for 3 years [33]. This discrepancy might imply complicated mechanisms between pharmacological metabolism of PPIs at the levels of cells and cognitive impairments presenting as a phenotype. Since the expression patterns are differed between diseases or from subject to subject, there may have been conflicting results regarding the effect of PPIs on cognitive impairments.

Both 1st-generation and 2nd-generation PPIs were associated with an increased likelihood for AD in the present study, which revealed that the associations between PPIs and AD were not different across PPI generations. Consistent with our findings, exposure to any PPI had a significantly increased risk of AD and any dementia [11, 12]. Different PPIs have resulted in elevated risks for dementia including omeprazole (hazard ratio 1.51; 95% CI = 1.40–1.64), pantoprazole (1.58; 95% CI = 1.40–1.79), and esomeprazole (2.12; 95% CI = 1.82–2.47) [12]. All 1st-generation and 2nd-generation PPIs had a similar negative impact on cognition after short-term exposure to PPIs [42]. Lansoprazole tended to slightly increase the relative risk of AD in the lag window models, although the authors concluded no association of specific PPI drug substances with AD risk [43]. Because both 1st-generation and 2nd-generation PPIs cross the blood–brain barrier, they are able to directly affect the brain [36, 38]. It is evident that all PPIs have some exacerbated effects on cognition [42].

### Strengths and limitations

The strength of this study is its use of a large, representative, nationwide population sample. To our knowledge, this is the largest nationwide nested case–control study to examine the association of PPI use with AD risk. Because the KNHIS-HSC data include all the hospitals and clinics of the entire nation without exception, no medical history was missed during the follow-up period. We comprehensively considered possible confounders. To minimize confounding effects, the control group was randomly selected by matching method.

Several limitations of the present study should be taken into account. The length of time for the analysis of PPI prescription, i.e., the 1-year period before the diagnosis of AD, can be considered short. We used prescription days, but actual medication intake could not be monitored in this study. We adjusted variables related to PPI use to minimize confounding effects between PPIs and AD; however, as it is a retrospective design, unmeasured confounding effects could not be completely excluded. Information on the family history of AD and genetic data, including apolipoprotein E4 allele status, was lacking in the health insurance data and was not taken into consideration.

## Conclusions

This nationwide population-based data may carefully indicate prior PPI use, regardless of current or past exposure, use duration, or use of 1st- or 2nd-generation PPIs, may increase the likelihood of AD in the > 60-year-old Korean population. Our results may provide supportive evidence regarding previous pharmacoepidemiologic studies of the potential negative impact of PPI on probability of AD.

## Supplementary Information

**Additional file 1: Supplementary 1**. Crude and adjusted odd ratios (95% confidence interval) of user of PPI (ref: non-user) for AD. **Supplementary 2**. Crude and adjusted odd ratios (95% confidence interval) of duration of PPI use (ref: 30 days versus ≥ 30 days) for AD. **Supplementary 3**. Crude and adjusted odd ratios (95% confidence interval) of the days of PPI use per 90 days by PPI generations for AD.

## Data Availability

All data are available from the database of National Health Insurance Sharing Service (NHISS) https://nhiss.nhis.or.kr/. NHISS allows access to all of this data for the any researcher who promises to follow the research ethics at some cost. If you want to access the data of this article, you can download it from the website after promising to follow the research ethics. Releasing of the data by the researcher is not allowed legally.

## Abbreviations

PPI: Proton pump inhibitor
AD: Alzheimer’s disease
KNHIS-HSC: Korean National Health Insurance Service-Health Screening Cohort data
ICD-10-CM: The International Classification of Diseases, 10th Revision, Clinical Modification
BMI: Body mass index
DBP: Diastolic blood pressure
SBP: Systolic blood pressure
CCI: Charlson Comorbidity Index
GERD: Gastroesophageal reflux disease
OR: Odds ratio
CI: Confidence interval
