# Supplementary material for: Associations between proton pump inhibitors and Alzheimer’s disease: a nested case–control study using a Korean nationwide health screening cohort
Source: Alzheimers Res Ther. 2022 Jul 1;14:91. doi: 10.1186/s13195-022-01032-5 (PMC9248149; doi:10.1186/s13195-022-01032-5)
Supplement: Supplementary file 1 — Additional file 1: Supplementary 1. Crude and adjusted odd ratios (95% confidence interval) of user of PPI (ref: non-user) for AD. Supplementary 2. Crude and adjusted odd ratios (95% confidence interval) of duration of PPI use (ref: 30 days versus ≥ 30 days) for AD. Supplementary 3. Crude and adjusted odd ratios (95% confidence interval) of the days of PPI use per 90 days by PPI generations for AD. [file 13195_2022_1032_MOESM1_ESM.docx]

**Supplementary Table 1** Crude and adjusted odd ratios (95% confidence interval) of user of PPI (ref: non-user) for AD

| Characteristics | | N of AD | N of Control | Odd ratios for AD | | *P*-value for  interaction |
| --- | --- | --- | --- | --- | --- | --- |
|  |  | (exposure/total, %) | (exposure/total, %) | Adjusted † | *P*-value |  |
| Age groups | | | | | | <0.001 |
|  | Age < 75 years old (n = 37,810) | 1,325/7,562 (17.5%) | 3,998/30,248 (13.2%) | 1.27 (1.18-1.37) | <0.001* |  |
|  | Age ≥ 75 years old (n = 48,315) | 1,591/9,663 (16.5%) | 5,463/38,652 (14.1%) | 1.15 (1.08-1.23) | <0.001* |  |
| Sex | | | | | | 0.607 |
|  | Male (n = 34,030) | 1,118/6,806 (16.4%) | 3,614/27,224 (13.3%) | 1.20 (1.11-1.30) | <0.001* |  |
|  | Female (n = 52,095) | 1,798/10,419 (17.3%) | 5,847/41,676 (14.0%) | 1.20 (1.13-1.28) | <0.001* |  |
| Income groups | | | | | | 0.356 |
|  | Low (n = 38,910) | 1,351/7,782 (17.4%) | 4,284/31,128 (13.8%) | 1.23 (1.14-1.32) | <0.001* |  |
|  | High (n = 47,215) | 1,565/9,443 (16.6%) | 5,177/37,772 (13.7%) | 1.18 (1.10-1.26) | <0.001* |  |
| Region of residence | | | | | | 0.228 |
|  | Urban (n = 30,030) | 918/6,006 (15.3%) | 3,084/24,024 (12.8%) | 1.12 (1.03-1.23) | 0.018* |  |
|  | Rural (n = 56,095) | 1,998/11,219 (17.8%) | 6,377/44,876 (14.2%) | 1.24 (1.16-1.31) | <0.001* |  |
| Obesity | | | | | | 0.682 |
|  | Underweight (n = 3,908) | 156/933 (16.7%) | 402/2,975 (13.5%) | 1.37 (1.09-1.71) | 0.007* |  |
|  | Normal (n = 32,056) | 1,136/6,921 (16.4%) | 3,363/25,135 (13.4%) | 1.20 (1.11-1.30) | <0.001* |  |
|  | Overweight (n = 21,469) | 691/4,082 (16.9%) | 2,392/17,387 (13.8%) | 1.15 (1.04-1.27) | 0.0055 |  |
|  | Obese (n = 28,692) | 933/5,289 (17.6%) | 3,304/23,403 (14.1%) | 1.21 (1.11-1.32) | <0.001* |  |
| Smoking status | | | | | | 0.539 |
|  | Non-smoker (n = 68,247) | 2,282/13,612 (16.8%) | 7,480/54,635 (13.7%) | 1.18 (1.12-1.25) | <0.001* |  |
|  | Past and current smoker (n = 17,878) | 634/3,613 (17.5%) | 1,981/14,265 (13.9%) | 1.24 (1.12-1.38) | <0.001* |  |
| Alcohol consumption | | | | | | 0.105 |
|  | <1 time a week (n = 64,651) | 2,146/13,381 (16.0%) | 6,707/51,270 (13.1%) | 1.17 (1.11-1.24) | <0.001* |  |
|  | ≥1 time a week (n = 21,474) | 770/3,844 (20.0%) | 2,754/17,630 (15.6%) | 1.28 (1.16-1.41) | <0.001* |  |
| Blood pressure | | | | | | 0.122 |
|  | SBP < 140 mmHg and DBP < 90 mmHg (n = 57,388) | 2,019/11,393 (17.7%) | 6,707/45,995 (14.6%) | 1.17 (1.11-1.25) | <0.001* |  |
|  | SBP ≥ 140 mmHg or DBP ≥ 90 mmHg (n = 28,737) | 897/5,832 (15.4%) | 2,754/22,905 (12.0%) | 1.26 (1.15-1.38) | <0.001* |  |
| Fasting blood glucose | | | | | | 0.625 |
|  | < 100 mg/dL (n = 48,895) | 1,545/9,292 (16.6%) | 5,366/39,603 (13.5%) | 1.21 (1.13-1.29) | <0.001* |  |
|  | ≥ 100 mg/dL (n = 37,230) | 1,371/7,933 (17.3%) | 4,095/29,297 (14.0%) | 1.18 (1.10-1.27) | <0.001* |  |
| Total cholesterol | | | | | | 0.871 |
|  | < 200 mg/dL (n = 47,433) | 1,673/9,345 (17.9%) | 5,481/38,088 (14.4%) | 1.21 (1.13-1.29) | <0.001* |  |
|  | ≥ 200 mg/dL (n = 38,692) | 1,243/7,880 (15.8%) | 3,980/30,812 (12.9%) | 1.17 (1.09-1.27) | <0.001* |  |
| CCI scores | | | | | | <0.001 |
|  | 0 (n = 44,558) | 888/6,254 (14.2%) | 4,581/38,304 (12.0%) | 1.18 (1.09-1.28) | <0.001* |  |
|  | 1 (n = 17,251) | 649/4,099 (15.8%) | 1,997/13,152 (15.2%) | 1.05 (0.94-1.16) | 0.402 |  |
|  | ≥2 (n = 24,316) | 1,379/6,872 (20.1%) | 2,883/17,444 (16.5%) | 1.26 (1.17-1.36) | <0.001* |  |
| GERD | | | | | | <0.001 |
|  | Absent (n = 70,316) | 1,278/13,958 (9.2%) | 3,602/56,358 (6.4%) | 1.32 (1.24-1.42) | <0.001* |  |
|  | Present (n = 15,809) | 1,638/3,267 (50.1%) | 5,859/12,542 (46.7%) | 1.14 (1.05-1.23) | 0.001* |  |
| H2-blocker | | | | | | <0.001 |
|  | Non-user (n = 26,086) | 294/4,432 (6.6%) | 1,479/21,654 (6.8%) | 1.01 (0.88-1.17) | 0.832 |  |
|  | User (n = 60,039) | 2,622/12,793 (20.5%) | 7,982/47,246 (16.9%) | 1.21 (1.15-1.28) | <0.001* |  |

Abbreviations: PPI, Proton pump inhibitor; AD, Alzheimer’s disease; SBP, Systolic blood pressure; DBP, Diastolic blood pressure; CCI, Charlson Comorbidity Index; GERD, Gastroesophageal reflux disease.

* Conditional or unconditional logistic regression analysis, Significance at *P* < 0.05

† The model was adjusted for age, sex, income, region of residence, for SBP, DBP, fasting blood glucose, total cholesterol obesity, smoking, alcohol consumption, CCI scores, GERD and H2-blocker.

**Supplementary Table 2** Crude and adjusted odd ratios (95% confidence interval) of duration of PPI use (ref: < 30 days versus ≥ 30 days) for AD

| Characteristics | | N of AD | N of Control | Odd ratios for AD | | *P*-value for  interaction |
| --- | --- | --- | --- | --- | --- | --- |
|  |  | (exposure/total, %) | (exposure/total, %) | Adjusted † | *P*-value |  |
| Age groups | | | | | | <0.001 |
|  | Age < 75 years old (n = 37,810) | 1,057/7,562 (14.0%) | 3,124/30,248 (10.3%) | 1.26 (1.16-1.37) | <0.001* |  |
|  | Age ≥ 75 years old (n = 48,315) | 1,221/9,663 (12.6%) | 4,198/38,652 (10.9%) | 1.14 (1.05-1.22) | <0.001* |  |
| Sex | | | | | | 0.991 |
|  | Male (n = 34,030) | 889/6,806 (13.1%) | 2,810/27,224 (10.3%) | 1.21 (1.11-1.33) | <0.001* |  |
|  | Female (n = 52,095) | 1,389/10,419 (13.3%) | 4,512/41,676 (10.8%) | 1.17 (1.09-1.26) | <0.001* |  |
| Income groups | | | | | | 0.174 |
|  | Low (n = 38,910) | 1,071/7,782 (13.8%) | 3,312/31,128 (10.6%) | 1.24 (1.14-1.34) | <0.001* |  |
|  | High (n = 47,215) | 1,207/9,443 (12.8%) | 4,010/37,772 (10.6%) | 1.15 (1.06-1.24) | <0.001* |  |
| Regoin of residence | | | | | | 0.554 |
|  | Urban (n = 30,030) | 713/6,006 (11.9%) | 2,316/24,024 (9.6%) | 1.13 (1.02-1.25) | 0.018* |  |
|  | Rural (n = 56,095) | 1,565/11,219 (13.9%) | 5,006/44,876 (11.2%) | 1.21 (1.14-1.30) | <0.001* |  |
| Obesity | | | | | | 0.365 |
|  | Underweight (n = 3,908) | 118/933 (12.6%) | 324/2,975 (10.9%) | 1.27 (0.98-1.64) | 0.067 |  |
|  | Normal (n = 32,056) | 898/6,921 (13.0%) | 2,607/25,135 (10.4%) | 1.21 (1.10-1.32) | <0.001* |  |
|  | Overweight (n = 21,469) | 512/4,082 (12.5%) | 1,830/17,387 (10.5%) | 1.09 (0.97-1.22) | 0.143 |  |
|  | Obese (n = 28,692) | 750/5,289 (14.2%) | 2,561/23,403 (10.9%) | 1.23 (1.12-1.35) | <0.001* |  |
| Smoking status | | | | | | 0.227 |
|  | Non-smoker (n = 68,247) | 1,764/13,612 (13.0%) | 5,775/54,635 (10.6%) | 1.16 (1.09-1.23) | <0.001* |  |
|  | Past and current smoker (n = 17,878) | 514/3,613 (14.2%) | 1,547/14,265 (10.8%) | 1.29 (1.15-1.46) | <0.001* |  |
| Alcohol consumption | | | | | | 0.095 |
|  | <1 time a week (n = 64,651) | 1,673/13,381 (12.5%) | 5,198/51,270 (10.1%) | 1.16 (1.08-1.23) | <0.001* |  |
|  | ≥1 time a week (n = 21,474) | 605/3,844 (15.7%) | 2,124/17,630 (12.0%) | 1.27 (1.14-1.42) | <0.001* |  |
| Blood pressure | | | | | | 0.048 |
|  | SBP < 140 mmHg and DBP < 90 mmHg (n = 57,388) | 1,570/11,393 (13.8%) | 5,205/45,995 (11.3%) | 1.15 (1.07-1.23) | <0.001* |  |
|  | SBP ≥ 140 mmHg or DBP ≥ 90 mmHg (n = 28,737) | 708/5,832 (12.1%) | 2,117/22,905 (9.2%) | 1.29 (1.17-1.43) | <0.001* |  |
| Fasting blood glucose | | | | | | 0.239 |
|  | < 100 mg/dL (n = 48,895) | 1,184/9,292 (12.7%) | 4,131/39,603 (10.4%) | 1.18 (1.09-1.27) | <0.001* |  |
|  | ≥ 100 mg/dL (n = 37,230) | 1,094/7,933 (13.8%) | 3,191/29,297 (10.9%) | 1.20 (1.10-1.30) | <0.001* |  |
| Total cholesterol | | | | | | 0.954 |
|  | < 200 mg/dL (n = 47,433) | 1,331/9,345 (14.2%) | 4,335/38,088 (11.4%) | 1.19 (1.11-1.28) | <0.001* |  |
|  | ≥ 200 mg/dL (n = 38,692) | 947/7,880 (12.0%) | 2,987/30,812 (9.7%) | 1.17 (1.07-1.27) | <0.001* |  |
| CCI scores | | | | | | <0.001 |
|  | 0 (n = 44,558) | 662/6,254 (10.6%) | 3,457/38,304 (9.0%) | 1.14 (1.03-1.26) | 0.009* |  |
|  | 1 (n = 17,251) | 505/4,099 (12.3%) | 1,544/13,152 (11.7%) | 1.04 (0.93-1.17) | 0.488 |  |
|  | ≥2 (n = 24,316) | 1,111/6,872 (16.2%) | 2,321/17,444 (13.3%) | 1.25 (1.15-1.36) | <0.001* |  |
| GERD | | | | | | <0.001 |
|  | Absent (n = 70,316) | 906/13,958 (6.5%) | 2,505/56,358 (4.4%) | 1.31 (1.21-1.42) | <0.001* |  |
|  | Present (n = 15,809) | 1,372/3,267 (42.0%) | 4,817/12,542 (38.4%) | 1.12 (1.04-1.22) | 0.004* |  |
| H2-blocker | | | | | | <0.001 |
|  | Non-user (n = 26,086) | 226/4,432 (5.1%) | 1,195/21,654 (5.5%) | 0.95 (0.81-1.12) | 0.544 |  |
|  | User (n = 60,039) | 2,052/12,793 (16.0%) | 6,127/47,246 (13.0%) | 1.21 (1.14-1.29) | <0.001* |  |

Abbreviations: PPI, Proton pump inhibitor; AD, Alzheimer’s disease; SBP, Systolic blood pressure; DBP, Diastolic blood pressure; CCI, Charlson Comorbidity Index; GERD, Gastroesophageal reflux disease.

* Conditional or unconditional logistic regression analysis, Significance at *P* < 0.05

† The model was adjusted for age, sex, income, region of residence, for SBP, DBP, fasting blood glucose, total cholesterol obesity, smoking, alcohol consumption, CCI scores, GERD and H2-blocker.

**Supplementary Table 3** Crude and adjusted odd ratios (95% confidence interval) of the days of PPI use per 90 days by PPI generations for AD

| Characteristics | | Mean (SD) of AD | Mean (SD) of Control | Odd ratios for AD | | *P*-value for  interaction |
| --- | --- | --- | --- | --- | --- | --- |
|  |  | (exposure/total, %) | (exposure/total, %) | Adjusted † | *P*-value |  |
| Age < 75 years old (n = 37,810) | | | | | | 1^st^-generation  : <0.001  2^nd^-generation  : <0.001 |
|  | The days of PPI prescription (1^st^-generation) | 10.25 (40.78) | 7.29 (34.16) | 1.10 (1.04-1.17) | 0.002* |  |
|  | The days of PPI prescription (2^nd^-generation) | 8.53 (40.01) | 5.30 (29.12) | 1.22 (1.14-1.30) | <0.001* |  |
| Age ≥ 75 years old (n = 48,315) | | | | | |  |
|  | The days of PPI prescription (1^st^-generation) | 10.46 (44.58) | 8.85 (41.34) | 1.05 (1.00-1.10) | 0.041* |  |
|  | The days of PPI prescription (2^nd^-generation) | 7.47 (37.34) | 6.87 (36.11) | 1.03 (0.97-1.09) | 0.298 |  |
| Male (n = 34,030) | | | | | | 1^st^-generation  : 0.052  2^nd^-generation  : 0.656 |
|  | The days of PPI prescription (1^st^-generation) | 10.18 (41.66) | 7.87 (37.30) | 1.08 (1.02-1.15) | 0.013* |  |
|  | The days of PPI prescription (2^nd^-generation) | 7.46 (36.98) | 5.83 (32.00) | 1.06 (1.01-1.11) | 0.009* |  |
| Female (n = 52,095) | | | | | |  |
|  | The days of PPI prescription (1^st^-generation) | 10.49 (43.77) | 8.36 (39.05) | 1.10 (1.02-1.18) | 0.015* |  |
|  | The days of PPI prescription (2^nd^-generation) | 8.25 (39.52) | 6.41 (34.01) | 1.10 (1.00-1.11) | 0.001* |  |
| Low income group (n = 38,910) | | | | | | 1^st^-generation  : 0.593  2^nd^-generation  : 0.859 |
|  | The days of PPI prescription (1^st^-generation) | 10.77 (43.95) | 8.17 (38.84) | 1.08 (1.02-1.14) | 0.006* |  |
|  | The days of PPI prescription (2^nd^-generation) | 8.44 (40.51) | 6.39 (34.23) | 1.12 (1.05-1.19) | <0.001* |  |
| High income group (n = 47,215) | | | | | |  |
|  | The days of PPI prescription (1^st^-generation) | 10.03 (42.11) | 8.16 (37.97) | 1.06 (1.00-1.11) | 0.034* |  |
|  | The days of PPI prescription (2^nd^-generation) | 7.52 (36.82) | 6.01 (32.38) | 1.08 (1.02-1.15) | 0.009* |  |
| Urban residents(n = 30,030) | | | | | | 1^st^-generation  : 0.597  2^nd^-generation  : 0.429 |
|  | The days of PPI prescription (1^st^-generation) | 9.03 (39.44) | 7.11 (35.27) | 1.06 (0.98-1.13) | 0.128 |  |
|  | The days of PPI prescription (2^nd^-generation) | 7.10 (36.37) | 5.62 (31.67) | 1.06 (0.98-1.14) | 0.137 |  |
| Rural residents (n = 56,095) | | | | | |  |
|  | The days of PPI prescription (1^st^-generation) | 11.08 (44.70) | 8.73 (39.91) | 1.07 (1.03-1.12) | 0.002* |  |
|  | The days of PPI prescription (2^nd^-generation) | 8.38 (39.64) | 6.48 (34.04) | 1.12 (1.06-1.17) | <0.001* |  |
| Underweight (n = 3,908) | | | | | | 1^st^-generation  : 0.553  2^nd^-generation  : 0.195 |
|  | The days of PPI prescription (1^st^-generation) | 11.70 (47.52) | 8.18 (37.58) | 1.21 (1.03-1.42) | 0.018* |  |
|  | The days of PPI prescription (2^nd^-generation) | 7.28 (35.58) | 6.25 (33.27) | 1.16 (0.94-1.42) | 0.160 |  |
| Normal weight (n = 32,056) | | | | | |  |
|  | The days of PPI prescription (1^st^-generation) | 10.28 (42.47) | 7.89 (37.60) | 1.07 (1.03-1.16) | 0.006* |  |
|  | The days of PPI prescription (2^nd^-generation) | 7.65 (37.65) | 5.94 (32.27) | 1.10 (1.03-1.18) | 0.005* |  |
| Overweight (n = 21,469) | | | | | |  |
|  | The days of PPI prescription (1^st^-generation) | 9.67 (41.06) | 7.76 (36.39) | 1.05 (0.97-1.13) | 0.254 |  |
|  | The days of PPI prescription (2^nd^-generation) | 7.62 (37.52) | 6.18 (33.09) | 1.04 (0.96-1.14) | 0.334 |  |
| Obese (n = 28,692) | | | | | |  |
|  | The days of PPI prescription (1^st^-generation) | 10.78 (44.14) | 8.76 (40.63) | 1.03 (0.97-1.10) | 0.290 |  |
|  | The days of PPI prescription (2^nd^-generation) | 8.67 (40.88) | 6.43 (34.33) | 1.12 (1.04-1.20) | 0.002* |  |
|  | The days of PPI prescription (2^nd^ generation) |  |  |  |  |  |
| Non-smoker (n = 68,247) | | | | | | 1^st^-generation  : 0.038  2^nd^-generation  : 0.342 |
|  | The days of PPI prescription (1^st^-generation) | 10.50 (43.94) | 8.19 (38.57) | 1.07 (1.03-1.11) | 0.002* |  |
|  | The days of PPI prescription (2^nd^-generation) | 7.57 (37.52) | 6.14 (33.22) | 1.07 (1.02-1.13) | 0.005* |  |
| Past and current smoker (n = 17,878) | | | | | |  |
|  | The days of PPI prescription (1^st^-generation) | 9.85 (39.01) | 8.09 (37.56) | 1.05 (0.96-1.15) | 0.258 |  |
|  | The days of PPI prescription (2^nd^-generation) | 9.31 (42.12) | 6.32 (33.27) | 1.18 (1.08-1.29) | <0.001* |  |
| Alcohol consumption < 1 time a week (n = 64,651) | | | | | | 1^st^-generation  : 0.067  2^nd^-generation  : 0.937 |
|  | The days of PPI prescription (1^st^-generation) | 10.41 (43.38) | 8.18 (38.55) | 1.07 (1.02-1.11) | 0.002* |  |
|  | The days of PPI prescription (2^nd^-generation) | 6.88 (35.87) | 5.50 (31.60) | 1.06 (0.98-1.15) | 0.008* |  |
| Alcohol consumption ≥ 1 time a week (n = 21,474) | | | | | |  |
|  | The days of PPI prescription (1^st^-generation) | 10.20 (41.41) | 8.13 (37.81) | 1.07 (1.02-1.13) | 0.156 |  |
|  | The days of PPI prescription (2^nd^-generation) | 11.61 (46.45) | 8.15 (37.51) | 1.15 (1.07-1.24) | <0.001* |  |
| SBP < 140 mmHg and DBP < 90 mmHg (n = 57,388) | | | | | | 1^st^-generation  : 0.040  2^nd^-generation  : 0.319 |
|  | The days of PPI prescription (1^st^-generation | 10.70 (43.45) | 8.44 (38.64) | 1.06 (1.01-1.11) | 0.011* |  |
|  | The days of PPI prescription (2^nd^-generation) | 8.22 (38.78) | 6.64 (34.13) | 1.08 (1.01-1.15) | 0.009* |  |
| SBP ≥ 140 mmHg or DBP ≥ 90 mmHg (n = 28,737) | | | | | |  |
|  | The days of PPI prescription (1^st^-generation) | 9.71 (41.95) | 7.61 (37.79) | 1.07 (1.02-1.13) | 0.025* |  |
|  | The days of PPI prescription (2^nd^-generation) | 7.38 (38.04) | 5.25 (31.32) | 1.16 (1.07-1.25) | <0.001* |  |
| Fasting blood glucose < 100 mg/dL (n = 48,895) | | | | | | 1^st^-generation  : 0.641  2^nd^-generation  : 0.370 |
|  | The days of PPI prescription (1^st^-generation) | 9.74 (41.26) | 7.93 (37.52) | 1.06 (1.00-1.11) | 0.033* |  |
|  | The days of PPI prescription (2^nd^-generation) | 7.43 (36.66) | 5.92 (32.06) | 1.07 (1.02-1.13) | 0.003* |  |
| Fasting blood glucose ≥ 100 mg/dL (n = 37,230) | | | | | |  |
|  | The days of PPI prescription (1^st^-generation) | 11.10 (44.84) | 8.49 (39.48) | 1.09 (1.03-1.16) | 0.012* |  |
|  | The days of PPI prescription (2^nd^-generation) | 8.52 (40.62) | 6.53 (34.75) | 1.10 (1.03-1.16) | 0.003* |  |
| Total cholesterol < 200 mg/dL (n = 47,433) | | | | | | 1^st^-generation  : 0.620  2^nd^-generation  : 0.696 |
|  | The days of PPI prescription (1^st^-generation) | 11.40 (44.82) | 9.01 (40.94) | 1.06 (1.01-1.11) | 0.016* |  |
|  | The days of PPI prescription (2^nd^-generation) | 8.66 (40.40) | 6.76 (34.91) | 1.09 (1.04-1.16) | 0.001* |  |
| Total cholesterol ≥ 200 mg/dL (n = 38,692) | | | | | |  |
|  | The days of PPI prescription (1^st^-generation) | 9.15 (40.59) | 7.13 (34.89) | 1.07 (1.01-1.14) | 0.028* |  |
|  | The days of PPI prescription (2^nd^-generation) | 7.08 (36.18) | 5.47 (31.02) | 1.10 (1.03-1.17) | 0.007* |  |
| CCI scores 0 (n = 44,558) | | | | | | 1^st^-generation  : <0.001  2^nd^-generation  : 0.001 |
|  | The days of PPI prescription (1^st^-generation) | 7.21 (35.56) | 6.22 (32.15) | 1.05 (0.98-1.14) | 0.153 |  |
|  | The days of PPI prescription (2^nd^-generation) | 6.42 (31.82) | 5.25 (29.45) | 1.09 (1.01-1.18) | 0.032* |  |
| CCI scores 1 (n = 17,251) | | | | | |  |
|  | The days of PPI prescription (1^st^-generation) | 8.97 (37.57) | 9.06 (40.15) | 1.09 (1.01-1.18) | 0.813 |  |
|  | The days of PPI prescription (2^nd^-generation) | 6.94 (34.87) | 7.06 (36.06) | 0.99 (0.90-1.09) | 0.858 |  |
| CCI scores ≥2 (n = 24,316) | | | | | |  |
|  | The days of PPI prescription (1^st^-generation) | 14.07 (51.07) | 11.78 (48.03) | 1.08 (1.03-1.14) | 0.003* |  |
|  | The days of PPI prescription (2^nd^-generation) | 9.91 (45.49) | 7.55 (38.37) | 1.13 (1.06-1.20) | <0.001* |  |
| GERD absent (n = 70,316) | | | | | | 1^st^-generation  : 0.399  2^nd^-generation  : 0.297 |
|  | The days of PPI prescription (1^st^-generation) | 5.21 (30.72) | 3.75 (27.16) | 1.09 (1.03-1.15) | 0.003* |  |
|  | The days of PPI prescription (2^nd^-generation) | 3.26 (25.47) | 2.38 (21.54) | 1.10 (1.03-1.18) | 0.005* |  |
| GERD present (n = 15,809) | | | | | |  |
|  | The days of PPI prescription (1^st^-generation) | 32.39 (71.39) | 28.02 (65.49) | 1.07 (1.02-1.12) | 0.008* |  |
|  | The days of PPI prescription (2^nd^-generation) | 27.91 (67.59) | 23.25 (60.20) | 1.11 (1.05-1.17) | <0.001* |  |
| H2-blocker non-user (n = 26,086) | | | | | | 1^st^-generation  : 0.002  2^nd^-generation  : <0.001 |
|  | The days of PPI prescription (1^st^-generation) | 4.40 (30.93) | 5.03 (33.49) | 0.95 (0.86-1.05) | 0.308 |  |
|  | The days of PPI prescription (2^nd^-generation) | 3.28 (27.36) | 3.51 (27.30) | 0.98 (0.88-1.10) | 0.765 |  |
| H2-blocker user (n = 60,039) | | | | | |  |
|  | The days of PPI prescription (1^st^-generation) | 12.43 (46.22) | 9.61 (40.32) | 1.09 (1.04-1.13) | <0.001* |  |
|  | The days of PPI prescription (2^nd^-generation) | 9.55 (41.60) | 7.40 (35.55) | 1.11 (1.06-1.16) | <0.001* |  |

Abbreviations: PPI, Proton pump inhibitor; AD, Alzheimer’s disease; SBP, Systolic blood pressure; DBP, Diastolic blood pressure; CCI, Charlson Comorbidity Index; GERD, Gastroesophageal reflux disease.

* Conditional or unconditional logistic regression analysis, Significance at *P* < 0.05

† The model was adjusted for age, sex, income, region of residence, for SBP, DBP, fasting blood glucose, total cholesterol obesity, smoking, alcohol consumption, CCI scores, GERD and H2-blocker.
